# Trends and potential human health risk of trace elements accumulated in transplanted blue mussels during restoration activities of Flekkefjord fjord (Southern Norway)

**DOI:** 10.1007/s10661-022-09835-7

**Published:** 2022-02-22

**Authors:** Marco Parolini, Sara Panseri, Federico Håland Gaeta, Luciana Rossi, Matteo Dell’Anno, Federica Ceriani, Beatrice De Felice, Trond Rafoss, Francesco Arioli, Salvatore Pilu, Luca Maria Chiesa

**Affiliations:** 1 Department of Environmental Science and Policy, University of Milan, via Celoria 26, 20133 Milan, Italy; 2 Department of Health, Animal Science and Food Safety, University of Milan, Via Celoria 10, 20133 Milan, Italy; 3 Norwegian Institute for Water Research (NIVA), N-4879 Grimstad, Norway; 4 Department of Natural Sciences, University of Agder (UiA), N-4630 Kristiansand, Norway; 5 Department of Agricultural and Environmental Sciences – Production, Land, Agroenergy, University of Milan, via Celoria 2, 20133 Milan, Italy

**Keywords:** Active biomonitoring, Bioaccumulation, Ecosystem restoration, Food safety

## Abstract

The monitoring of contaminants represents a priority to preserve the integrity of marine ecosystems, as well as to plan and to manage restoration activities in order to protect environmental and human health. In the present study, a 6-months active biomonitoring was performed to explore the levels of eighteen trace and toxic elements, including heavy metals (TEs; i.e. Al, As, Ca, Cd, Cr, Cu, Fe, Hg, K, Mg, Mn, Na, Ni, P, Pb, Sr, Ti, and Zn), accumulated in soft tissues of blue mussel (*Mytilus edulis* Linnaeus, 1758) individuals transplanted at different depths (5- and 15-m depth) in five locations within the Flekkefjord fjord (Southern Norway). As this area suffered a long-lasting contamination due to both organic and inorganic contaminants, a series of restoration activities were activated to tackle and to prevent potential risks for ecosystem and local population. Our results demonstrated that the levels of TEs accumulated in edible tissues of transplanted mussels in the Flekkefjord fjord were generally low before the beginning of the restoration activities. However, location- and time-specific differences in the accumulation of TEs were noted after the implementation of such activities. Interestingly, the levels of Fe and Mn significantly increased after the beginning of the restoration activities, likely because the release of these TEs from the slag used in such operations and/or resuspension of contaminated sediments. However, assuming that native mussels can accumulate the same TEs at levels measured in transplanted individuals, our results suggest a substantial safety for human consumption of native mussels from the Flekkefjord fjord, regardless of restoration activities.

## Introduction

Marine ecosystems represent a sink of diverse anthropogenic-derived inorganic and organic contaminants. Among them, trace elements (TEs) have been recognized for a long time as globally distributed contaminants and a serious threat for environmental and human health (Abbasi et al., 2015). Trace elements are non-biodegradable inorganic contaminants originating by both natural geological and anthropogenic processes (Hejna et al., 2018). Some TEs play a crucial role in diverse biological functions (e.g. zinc, copper and iron) or return positive effects to the organism even in small quantities (e.g. manganese or nickel), contributing to maintain a good health status in humans and animals (Vandermeersch et al., 2015). However, some findings suggest that the use of zinc, copper, and metals in animal nutrition represents a risk factor for methicillin-resistant *Staphylococcus aureu*s (MRSA), as these compounds are associated with the co-selection of resistance genes to antibiotic (Seiler & Berendonk, 2012). Other TEs have no biological role and can cause toxic effects or be potentially lethal also at low concentrations (e.g. mercury, lead, cadmium, arsenic; Aras & Ataman, 2007). In addition, some TEs can accumulate along the trophic chain (Bostan et al., 2007; Burger & Gochfeld, 2004) and induce detrimental effects at different levels of the ecological hierarchy, including humans.

In marine ecosystems, bottom sediments represent the main sink of several contaminants, including TEs, which can be accumulated by benthic organisms or released back to the water (Pan & Wang, 2012). Different processes can cause the resuspension to the overlying water of sediment-associated contaminants, such as the variation in physical and chemical environmental variables (e.g. pH, salinity, redox potential), natural phenomena caused by waves, currents and bioturbation and/or anthropic disturbances (e.g. boat wash, bottom trawling, dredging and disposal activities; Hedman et al., 2009; Jonas & Millward, 2010; Juwarkar et al., 2010). For instance, periodical dredging activities are regularly performed for preserving the navigation in harbours and/or for restoration purposes of contaminated ecosystems, leading to the resuspension of contaminated sediments and the increase of contaminant bioavailability. Thus, sediment resuspension can represent a serious hazard for the health of marine organisms and, potentially, of humans consuming contaminated seafood. In fact, long-term exposure and/or high concentrations of some TEs can induce a series of detrimental effects on human health, ranging from skin diseases to nervous system, blood and gastrointestinal dysfunctions, respiratory problems, and mutagenic and carcinogenic effects (Martin & Griswold, 2009).

Marine molluscs are considered as good ‘sentinels’ to monitor the levels and the trends of organic and inorganic contaminants. Their filter-feeding behaviour and sessile habit allow them the accumulation of contaminants in soft tissues proportionally to levels in seawater (Fiorito et al., 2019; Grbin et al., 2019). The use of mussels, including native or transplanted individuals from an unpolluted area, represents a suitable strategy for biomonitoring spatial and temporal trends of contamination of coastal waters (Abderrahmani et al., 2021; Bajt et al., 2019; Esposito et al., 2021; Parolini et al., 2020a). Moreover, mussels can be useful to check for the effectiveness of restoration activities and to assess the environmental risk due to contaminant exposure by comparing measured levels with quality standards or regulatory benchmarks (Beyer et al., 2017; Nekhoroshkov et al., 2021; Parolini et al., 2020a). Several field studies have demonstrated the reliability of mussels to monitor the levels of TEs in different ecosystems worldwide (e.g. Farrington et al. 2016; Schøyen et al., 2017; Krishnakumar et al., 2018; Abderrahmani et al., 2021; Greggio et al., 2021; Esposito et al., 2021). Similarly, the biomonitoring using mussels confirmed that dredging operations play a crucial role in resuspension of contaminated sediments and subsequent uptake of heavy metals (e.g. Bocchetti et al., 2008). In addition, because of the importance of mussels as seafood for humans, monitoring the levels of TEs accumulated in their tissues can be used to assess potential health risk due to mussel consumption through the comparison of measured TEs levels with consumer safety thresholds set by the national or international regulations (Beyer et al., 2017; Chiesa et al., 2018).

The present active biomonitoring study aimed at measuring the levels of eighteen TEs (i.e. Al, As, Ca, Cd, Cr, Cu, Fe, Hg, K, Mg, Mn, Na, Ni, P, Pb, Sr, Ti, and Zn) accumulated over a 6-months period in soft tissues of blue mussel *Mytilus edulis* (Linnaeus, 1758) individuals transplanted in five locations within the Flekkefjord fjord (Southern Norway). In addition to well-known toxic metals (e.g. Hg, Pb, Cr, and Cu), we focused also on the levels and trends of essential TEs because some studies have demonstrated that the excess of essential elements, such as Mn, Mg, and K, can be detrimental for mussel species (e.g. Archambault et al., 2017; Gillis et al., 2021; Kleinhenz et al., 2019). This area suffered a long-lasting input of organic (e.g. PCBs and PAHs) and inorganic (i.e. heavy metals) contaminants due to industrial and naval activities, as well as to the input of municipal waste (Misund, 2012). To tackle for the contamination and to prevent potential risks for ecosystem and human health, in August 2018 a series of restoration activities, including the dredging of bottom sediments and the covering of the seabed with sand to isolate any residual of contamination, were implemented. Previous studies on mussels (Parolini et al., 2020a) and demersal fish (Parolini et al., 2020b) showed an increase in organohalogen compounds after the beginning of restoration activities, with potential human health risk due to mussel consumption (Parolini et al., 2020a). As to date no information is available for TEs, the main goals of this study were the monitoring of TEs levels and trends before and during the sediment restoration activities, as well as the assessment of potential health risk due to human consumption of contaminated mussels.

## Materials and methods

### Active biomonitoring

**Fig. 1:**
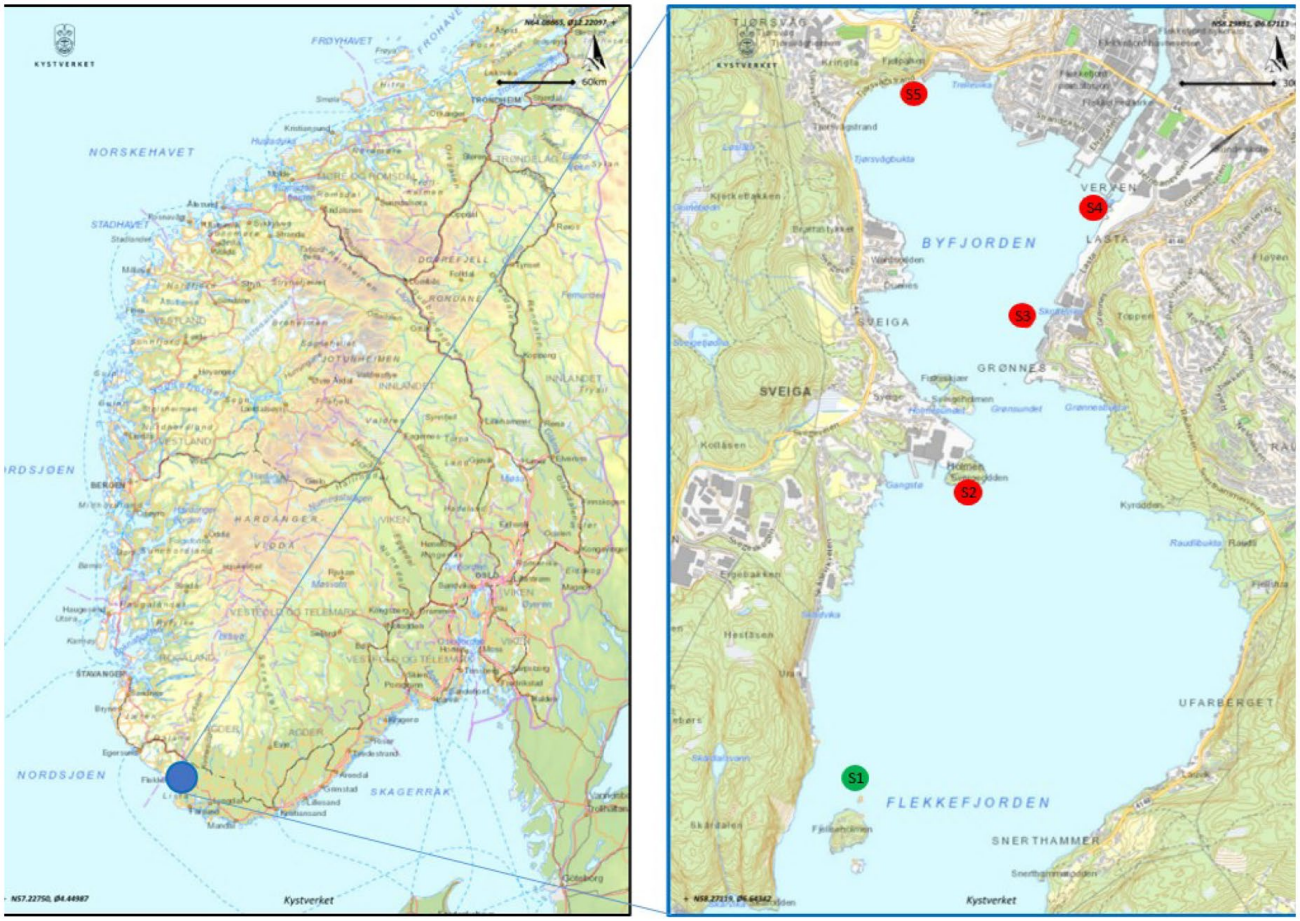
Geographical localization of the mussel cages in Lafjord and Byfjorden in Flekkefjord fjord, Southern Norway

The active biomonitoring was performed in the Flekkefjord fjord over a 6-months period in 2018, from June the 27th and December the 15th. The experimental design of biomonitoring survey was detailed by Parolini et al. (2020a). Briefly, blue mussel individuals (3–5 cm in length) were purchased from a mussel farm located in Kaldvellfjord (Lillesand, Norway), which is far from local sources of contamination (Schøyen et al., 2017). Mussels were transplanted in cages to five locations (S1—S5; Fig. 1), which were identified according to the levels of TEs, mainly heavy metals, measured in sediments by previous monitoring surveys (Haker, 2011; Misund, 2012).

The caging location 1 (S1; 58° 16′ 30.0″ N—6° 39′ 12.9″ E) was located in the outer part of Flekkefjord fjord and was chosen as a reference site, while the caging locations S2–S5 were close to the areas where sediment restoration activities were implemented. In detail, S2 (58° 17′ 02.7″ N—6° 39′ 15.6″ E) and S3 (58° 17′ 23.0″ N—6° 39′ 30.9″ E) were located nearby an old ship industry and the old industrial area called ‘Slippen’, respectively. The sampling locations S4 (58° 17′ 33.8″ N—6° 39′ 41.3″ E) and S5 (58° 17′ 43.3″ N—6° 39′ 12.5″ E) were located close to an old landfill and an abandoned tannery, respectively. Two cages containing approximatively 300 mussels each were placed using buoys, ropes, and weights by a scuba diver in the five selected locations at 5- and 15-m depth. The active biomonitoring survey began on June the 27th (*t* = 0 days), and soon after the placement of cages in water, about 50 specimens from each cage were sampled to check for background levels of trace elements. Sampling of about 50 mussels per each cage was then performed on July the 27th (*t* = 30 days), before the beginning of restoration activities that started on August 2018, October the 10th (*t* = 135 days), November the 15th (*t* = 166 days), and December the 15th (*t* = 196 days) to follow the trend of TE contamination over a 6-months period of time. After sampling, mussels were transported to the lab within 1 h, their soft tissues were separated from the shells and pooled in a unique sample per sampling location (S1–S5), depth (5 and 15 m), and time (*t* = 0 – t = 196 days). Each pool was weighed to determine the wet weight and stored at −20 °C until processing. The mussels were not depurated before dissection. Mussels were lyophilized in a freeze dryer (Edwards Pirani 1001) for about 36 h. After dry weight determination, mussel tissues were ground and stored into glass bottles within a desiccator pending analysis of TEs. Mussels transplanted in S1 and S2 could not be collected after *t* = 166 days because coastal storms wiped out the cages, precluding the analysis of trace elements in these locations at *t* = 166 days and *t* = 196 days. The sampling in S5 at *t* = 196 days was not performed because all the mussels had died probably as the consequence of the undersea landslide that occurred after *t* = 135 days close to this location.

### Analysis of trace elements

Mussels from each sampling were dried and ground through a 1 mm grid screen in order to obtain a homogeneous sample. A total of 0.3 g of each sample was mineralized by an ultrawave single reaction chamber microwave digestion system (Anton Paar MULTIWAVE 3000, Anton Paar GmbH, Graz, Austria) in Teflon tubes filled with 10 ml of HNO_3_ (65% concentrated) by applying a one-step temperature ramp (at 120 °C in 10 min and maintained for 10 min). The mineralized samples were cooled for 20 min and the homogenous sample solutions were transferred into polypropylene test tubes. Samples (250 μL) were then diluted 1:40 (v/v) with a standard solution containing an internal standard (100 μL) and H_2_O (9.75 mL) according to Hejna et al. (2020). In particular, an aliquot of 2 mg/L of an internal standard solution (^72^Ge, ^89^Y, ^159^ Tb) was added to the samples and calibration curve to obtain a final concentration of 20 mg/L. The accuracy and precision of the results obtained using ICP-MS were evaluated using Oyster Tissue as reference materials (Reference Material^®^ 1566b, National Institute of Standards and Technology, Gaithersburg, MD, USA). All samples were analysed in triplicate by inductively coupled plasma mass spectrometry (ICP-MS; Bruker Aurora M90 ICP-MS, Bremen, Germany) in order to detect the following trace elements: Al, As, Ca, Cd, Cr, Cu, Fe, Hg, K, Mg, Mn, Na, Ni, P, Sr, Ti, Pb, and Zn. The typical polyatomical analysis interferences were removed using the collision-reaction interface (CRI) with an H_2_ flow of 75 mL/min through a skimmer cone. Each sample was analysed in duplicate. TE concentrations were first calculated on dry weight (C_dw_) basis and then converted to wet weight (C_ww_) basis according to the following formula: C_ww_ = C_dw_ × [(100 − % moisture)/100]. Concentrations of TEs were reported on wet weight basis for each sample and sampling time (Table 1).

**Table 1:** Concentrations of TEs (expressed as mg/kg ww) measured in soft tissues of mussels transplanted in five locations (S1–S5) within the Flekkefjord fjord over a 6-months active biomonitoring activity

Location	Depth (m)	Sampling (days)	Al	As	Ca	Cd	Cr	Cu	Fe	Hg	K	Mg	Mn	Na	Ni	P	Pb	Sr	Ti	Zn
	5	*t* = 0	178.06	2.00	909.13	0.20	0.20	2.60	67.09	< 0.001	1,559.57	1,048.01	2.51	4,159.59	0.04	1,192.57	0.72	8.55	1.63	12.87
	15	*t* = 0	92.34	1.73	2,357.47	0.18	0.34	3.01	75.40	0.001	2,616.83	2,576.50	2.69	9,665.28	0.18	2,181.46	0.84	14.43	2.17	18.90
	5	*t* = 30	70.38	1.87	469.32	0.19	0.30	1.13	27.88	< 0.001	1,873.53	426.20	0.85	3,958.54	0.21	1,003.90	0.99	6.41	0.54	7.68
	15	*t* = 30	126.20	1.68	2,379.86	0.17	0.19	2.78	42.19	< 0.001	1,744.09	948.16	1.12	5,193.29	0.35	871.11	0.59	10.93	1.37	15.03
**S1**	5	*t* = 135	65.59	1.48	550.90	0.18	0.18	2.50	40.17	0.001	1,450.62	671.00	0.97	3,621.76	0.22	775.25	0.66	5.85	1.44	12.96
	15	*t* = 135	43.00	1.66	2,450.64	0.17	0.37	5.83	35.01	0.001	1,483.59	607.64	1.52	3,723.08	0.22	804.01	0.82	< LOD	0.82	17.34
	5	*t* = 166	142.41	1.52	887.46	0.18	0.30	2.49	39.37	< 0.001	1,048.56	674.76	0.99	2,618.14	0.43	906.93	0.65	6.94	1.13	12.70
	15	*t* = 166	206.45	1.66	1,444.78	0.15	0.21	2.14	39.99	< 0.001	1,042.82	823.62	1.56	3,141.89	0.39	945.70	0.54	9.84	1.07	11.87
	5	*t* = 196	103.56	1.69	6,961.05	0.16	0.29	2.45	42.06	0.001	1,023.53	1,046.80	2.40	3,306.93	0.46	1,011.55	0.72	< LOD	1.07	15.72
	15	*t* = 196	73.49	1.64	1,584.19	0.21	0.26	2.82	35.79	< 0.001	1,363.39	634.97	1.06	2,982.82	0.23	924.98	0.72	8.40	1.12	17.22
	5	*t* = 0	13.94	2.33	920.71	0.19	0.24	0.75	23.45	0.001	1,521.35	1,235.21	0.97	4,110.97	0.18	1,345.11	0.80	10.56	0.47	6.34
	15	*t* = 0	19.98	2.85	434.68	0.30	0.10	1.49	36.70	0.001	735.78	406.97	1.24	1,602.02	0.30	563.03	0.42	4.46	1.18	6.77
	5	*t* = 30	17.29	2.40	675.82	0.14	0.19	5.78	16.65	< 0.001	3,853.87	2,314.08	0.96	11,481.13	0.09	1,118.33	0.42	< LOD	0.29	12.06
	15	*t* = 30	33.55	2.68	792.25	0.14	0.17	1.69	32.28	< 0.001	1,869.64	1,678.54	1.02	7,049.09	0.30	1,353.87	0.47	8.13	0.92	10.22
**S2**	5	*t* = 135	18.52	2.48	719.42	0.17	0.29	0.79	31.42	< 0.001	1,246.48	1,191.81	1.09	3,531.01	0.24	1,318.25	0.75	12.33	0.67	5.77
	15	*t* = 135	28.79	1.84	856.86	0.14	0.34	1.42	61.97	0.001	1,215.77	1,297.62	1.57	4,382.85	0.27	1,386.15	0.54	8.78	1.54	10.95
	5	*t* = 166	25.25	2.78	847.06	0.16	0.29	1.03	40.77	< 0.001	2,460.38	2,013.35	1.29	7,886.65	0.26	1,289.49	0.62	11.75	1.00	7.16
	15	*t* = 166	17.52	2.00	870.78	0.11	0.24	0.79	34.15	0.001	2,136.33	1,696.56	1.59	6,308.51	0.15	1,352.45	0.41	6.76	0.67	5.73
	5	*t* = 196	13.44	2.72	1,914.48	0.17	0.23	3.10	52.03	0.001	3,350.05	2,588.45	1.53	10,170.42	0.51	1,136.89	0.57	19.46	1.57	15.34
	15	*t* = 196	21.50	2.36	1,478.43	0.22	0.31	2.81	56.45	< 0.001	1,162.87	1,194.09	1.87	2,861.15	0.34	1,469.68	0.79	12.87	1.35	11.54
	5	*t* = 0	31.23	1.47	6,540.06	0.11	0.52	2.62	61.94	0.001	1,450.88	1,249.26	6.70	5,014.48	0.41	1,112.78	0.82	20.72	1.84	15.96
	15	*t* = 0	29.39	1.83	2,355.60	0.21	0.48	2.23	56.51	0.003	1,727.97	1,824.31	2.87	6,386.65	0.28	1,187.22	0.49	13.86	1.56	21.14
	5	*t* = 30	22.40	1.65	1,039.33	0.18	0.39	2.03	47.50	0.001	1,184.01	1,181.46	4.63	3,927.58	0.07	1,174.21	0.55	8.35	1.28	17.23
	15	*t* = 30	n.a	n.a	n.a	n.a	n.a	n.a	n.a	n.a	n.a	n.a	n.a	n.a	n.a	n.a	n.a	n.a	n.a	n.a
**S3**	5	*t* = 135	99.49	2.19	3,210.63	0.19	0.38	3.72	61.11	0.002	1,541.34	1,454.19	34.16	4,178.84	0.68	1,403.41	0.91	27.02	1.89	14.05
	15	*t* = 135	50.26	1.60	4,212.12	0.17	0.42	1.18	47.17	0.001	1,345.68	1,843.20	25.28	4,637.78	0.43	1,363.72	0.69	< LOD	1.10	21.48
	5	*t* = 166	124.68	1.87	2,924.16	0.22	0.45	3.40	73.64	< 0.001	1,974.07	1,847.19	72.17	5,719.47	0.33	1,127.24	0.64	20.19	2.62	20.70
	15	*t* = 166	76.83	1.42	1,761.83	0.14	0.41	3.43	61.62	< 0.001	1,341.25	990.45	36.52	2,666.12	0.13	1,413.63	0.94	12.67	2.35	14.52
	5	*t* = 196	100.29	1.69	1,189.94	0.16	0.32	1.19	63.04	0.001	1,676.21	1,453.86	55.33	4,977.90	0.31	1,218.74	0.59	11.39	1.84	17.52
	15	*t* = 196	44.35	1.82	880.40	0.15	0.30	2.60	44.99	< 0.001	2,074.24	1,629.99	40.48	6,879.07	0.31	1,168.37	0.77	9.74	1.32	22.30
	5	*t* = 0	n.a	n.a	n.a	n.a	n.a	n.a	n.a	n.a	n.a	n.a	n.a	n.a	n.a	n.a	n.a	n.a	n.a	n.a
	15	*t* = 0	n.a	n.a	n.a	n.a	n.a	n.a	n.a	n.a	n.a	n.a	n.a	n.a	n.a	n.a	n.a	n.a	n.a	n.a
	5	*t* = 30	n.a	n.a	n.a	n.a	n.a	n.a	n.a	n.a	n.a	n.a	n.a	n.a	n.a	n.a	n.a	n.a	n.a	n.a
	15	*t* = 30	n.a	n.a	n.a	n.a	n.a	n.a	n.a	n.a	n.a	n.a	n.a	n.a	n.a	n.a	n.a	n.a	n.a	n.a
**S4**	5	*t* = 135	636.38	1.78	890.87	0.15	0.17	1.14	31.33	0.001	1,414.67	1,401.97	6.83	4,714.08	0.35	1,332.87	0.55	8.93	0.63	7.27
	15	*t* = 135	158.27	1.44	6,493.02	0.12	0.39	3.88	69.43	0.001	1,251.00	1,718.72	16.38	3,159.98	0.45	1,460.17	1.18	< LOD	2.02	19.24
	5	*t* = 166	75.72	1.93	1,226.32	0.16	0.22	2.85	53.25	< 0.001	1,686.65	1,365.07	6.13	4,574.64	0.33	1,255.46	0.65	10.68	1.63	14.44
	15	*t* = 166	115.03	1.70	2,849.24	0.15	0.35	2.92	57.15	0.002	1,488.75	1,479.27	11.59	3,741.05	0.22	1,607.97	0.82	23.86	1.71	17.39
	5	*t* = 196	23.48	0.28	2,136.83	0.28	0.19	2.43	43.77	0.001	1,401.49	1,330.90	11.51	4,964.83	0.28	1,233.98	0.57	11.13	1.57	14.68
	15	*t* = 196	41.24	1.86	952.20	0.18	0.24	2.25	48.34	< 0.001	1,496.60	1,837.52	35.96	5,749.39	0.49	1,493.25	0.48	9.74	1.33	25.55
	5	*t* = 0	n.a	n.a	n.a	n.a	n.a	n.a	n.a	n.a	n.a	n.a	n.a	n.a	n.a	n.a	n.a	n.a	n.a	n.a
	15	*t* = 0	n.a	n.a	n.a	n.a	n.a	n.a	n.a	n.a	n.a	n.a	n.a	n.a	n.a	n.a	n.a	n.a	n.a	n.a
	5	*t* = 30	22.30	3.50	708.30	0.13	0.24	0.66	45.91	0.001	907.59	803.37	2.05	2,888.57	0.37	1,786.55	0.77	7.26	0.51	15.37
	15	*t* = 30	n.a	n.a	n.a	n.a	n.a	n.a	n.a	n.a	n.a	n.a	n.a	n.a	n.a	n.a	n.a	n.a	n.a	n.a
**S5**	5	*t* = 135	29.12	2.24	909.14	0.10	0.27	2.26	50.60	0.001	1,173.97	1,071.39	6.10	4,107.22	0.26	1,872.78	0.85	9.54	1.47	18.57
	15	*t* = 135	16.93	2.07	1,577.68	0.13	0.34	3.30	58.94	< 0.001	1,681.48	1,467.16	18.13	5,698.39	0.33	1,370.38	0.45	< LOD	0.63	21.32
	5	*t* = 166	32.47	2.15	3,547.40	0.13	0.37	2.49	67.88	< 0.001	1,285.23	1,309.62	6.81	2,723.69	0.26	1,510.00	0.76	< LOD	1.85	19.10
	15	*t* = 166	21.26	2.05	1,685.58	0.12	0.26	2.16	72.92	0.001	2,074.41	1,731.46	22.44	7,279.30	0.26	1,383.87	0.51	12.65	1.44	20.76
	5	*t* = 196	29.68	2.56	2,697.95	0.10	0.30	3.08	64.79	0.001	1,532.33	1,406.36	11.98	4,040.69	0.39	1,553.11	0.88	21.27	1.99	16.84
	15	*t* = 196	n.a	n.a	n.a	n.a	n.a	n.a	n.a	n.a	n.a	n.a	n.a	n.a	n.a	n.a	n.a	n.a	n.a	n.a

*< LOD*, below the limit of detection. *n.a.*, not available because the mussel sample was missing

### Human health risk assessment

The human health risk assessment for potential mussel consumption relied on two different approaches. First, direct comparisons of levels measured in the edible part of mussels with safety guidelines set by the Commission Regulation (EC) No 1259/2011 and based on established maximum permissible limits (MPLs) were performed for Cd, Hg, and Pb only. The second approach relied on the calculation of target hazard quotient (THQ), representing the ratio between the exposure and a reference dose estimating the daily exposure to the human population that does not result in a considerable risk of deleterious effects during its lifetime (Perošević et al., 2018 and references therein). For each trace element, the THQ was calculated according to the following equation (USEPA, 2005):

$$\mathrm{THQ }=\frac{\mathrm{EF }\times \mathrm{ED }\times \mathrm{ MS }\times \mathrm{C}}{RfDo \times BW \times AT} \times {10}^{-3}$$

whereby EF represents the exposure frequency (350 days/year, representing an exposure reasonably expected in a location with 2 weeks of vacation or travel; USEPA, 2005); ED is the exposure duration in terms of average human lifetime (70 years); MS is a mussel meal size (2.76 g/capita/day calculated by FAOSTAT for molluscs in the Norwegian population; FAOSTAT, 2015); C is the concentration of a trace element in the edible portion of mussels (expressed in mg/kg wet weight); RfDo is an oral reference dose (mg/kg of body weight per day) provided by the USEPA (2017) and listed by Perošević et al. (2018); BW is a body weight of an adult (i.e. 70 kg) and AT is an averaging time (ED × 365 days/year). THQ was calculated for trace elements whose RfDo was available, namely Al, Cd, Cr, Cu, Fe, Mn, Ni, Pb, Sr, and Zn. As RfDo for Hg was not available, we used the value calculated for methyl-Hg (MeHg).

For the risk assessment of multiple trace elements accumulated in mussels, a total hazard index (HI) was estimated to evaluate an overall risk from different elements. The HI was calculated according to the following formula (USEPA, 2005):

$$\mathrm{HI}=\sum \limits_ {i=1}^{n}\mathrm{THQ}i$$

where THQ*i* is the THQ calculated as above for each single trace element and *n* represents the number of elements considered in the health risk assessment (*n* = 14). Whenever the HI value exceeds the unit, a concern for potential health effects might occur (Perošević et al., 2018).

## Statistical analysis

General linear models (GLM) including sampling Location, Time, and Depth as factors, and their two-way interactions, were run to investigate changes in levels of TEs accumulated in soft tissues of transplanted mussels. As Location × Depth interaction did not return any significant effects for all the TEs and Time × Depth interaction returned only spurious effects for K, Mn, and Fe, they were removed from the final model in a single step. Statistical analyses were run in R 3.6.1 (R Core Team, 2019).

## Results

The survival of caged mussels during the 6-months biomonitoring was high, although the health status of mussels transplanted in S1 and S2 could not be monitored after *t* = 166 days because the cages disappeared. In addition, the cage placed at 15-m depth in S3 was plundered by crabs after the third sampling (*t* = 166 days); thus, we collected less than 50 mussels (~ 20 individuals) at *t* = 166 and 196 days. Complete mortality of mussels occurred at *t* = 166 days in the cage placed at 15-m depth in S5, precluding the sampling of organisms at *t* = 196 days. Although all the mussels died, as their soft tissue was inside the shells, a sample was collected for TE analyses to assess their potential contribution to mussel death.

Levels of TEs (expressed as mg/kg wet weight—ww) measured in blue mussels transplanted in the five locations of the Flekkefjord fjord over 6-months biomonitoring activity are reported in Table 1. Overall, the mean (± SE; min–max) concentrations of essential elements measured in mussel soft tissues, such as Na (4,872.80 ± 332.36; 1,602.02–11,481.13 mg/kg ww), Ca (1,934.00 ± 253.79; 434.68–6,961.05 mg/kg ww), K (1,621.19 ± 94.19; 735.78–3,853.87 mg/kg ww), Mg (1,352.95 ± 81.76; 406.97–2,588.45 mg/kg ww), and P (1,267.81 ± 47.40; 563.03–2,181.46 mg/kg ww), were higher compared to the other TEs, independently of sampling location and depth. However, their concentrations did not significantly differ among sampling locations, times of sampling, and depths. In fact, GLM did not show any significant effect of the main factors or their two-way interactions (Table 2). Levels (estimated marginal means ± standard error) of other TEs were generally one order of magnitude lower compared to those mentioned above. Al (75.42 ± 16.03; 13.44–636.38 mg/kg ww) was the more abundant TE measured in mussel tissues, followed by Fe (49.09 ± 2.29; 16.65–75.40 mg/kg ww), Zn (14.87 ± 0.79; 5.73–25.55 mg/kg ww), Mn (11.29 ± 2.58; 0.85–72.17 mg/kg ww), Sr (9.89 ± 1.04; < LOQ–27.02 mg/kg ww), Cu (2.42 ± 0.18; 0.66–5.83 mg/kg ww), As (1.96 ± 0.08; 0.28–3.50 mg/kg ww), and Ti (1.33 ± 0.08; 0.29–2.62 mg/kg ww). The levels of the most hazardous TEs, such as Pb (0.68 ± 0.03; 0.41–1.18 mg/kg ww), Ni (0.30 ± 0.02; 0.04–0.68 mg/kg ww), Cr (0.29 ± 0.01; 0.10–0.52 mg/kg ww), Cd (0.17 ± 0.01; 0.10–0.30 mg/kg ww), and Hg (0.0007 ± 0.0001; < 0.0001–0.0025 mg/kg ww), were negligible compared to the other ones.

**Table 2:** Effect of sampling location, time, and depth, as well as their two-way interactions, obtained by general linear models (GLM) on the levels of trace elements measured in mussels transplanted to the Flekkefjord fjord

Trace element	Location	Time	Depth	Location × Time
Al	F_4,40_ = 0.940; *P* = 0.479	F_4,40_ = 3.942; *P* = **0.035**	F_1,40_ = 1.296; *P* = 0.281	F_13,40_ = 1.596; *P* = 0.232
As	F_4,40_ = 1.742; *P* = 0.217	F_4,40_ = 10.788; *P* = **0.001**	F_1,40_ = 0.510; *P* = 0.491	F_13,40_ = 1.177; *P* = 0.405
Ca	F_4,40_ = 0.866; *P* = 0.517	F_4,40_ = 1.725; *P* = 0.221	F_1,40_ = 0.007; *P* = 0.936	F_13,40_ = 1.301; *P* = 0.343
Cd	F_4,40_ = 2.327; *P* = 0.127	F_4,40_ = 2.281; *P* = 0.132	F_1,40_ = 0.071; *P* = 0.795	F_13,40_ = 1.330; *P* = 0.330
Cr	F_4,40_ = 1.936; *P* = 0.181	F_4,40_ = 11.067; *P* = **0.001**	F_1,40_ = 0.673; *P* = 0.431	F_13,40_ = 1.256; *P* = 0.364
Cu	F_4,40_ = 0.177; *P* = 0.945	F_4,40_ = 0.595; *P* = 0.675	F_1,40_ = 0.485; *P* = 0.502	F_13,40_ = 1.274; *P* = 0.356
Fe	F_4,40_ = 7.677; *P* = **0.004**	F_4,40_ = 12.877; *P* < **0.001**	F_1,40_ = 3.073; *P* = 0.110	F_13,40_ = 4.112; *P* = **0.016**
Hg	F_4,40_ = 1.056; *P* = 0.427	F_4,40_ = 1.329; *P* = 0.325	F_1,40_ = 0.225; *P* = 0.645	F_13,40_ = 0.815; *P* = 0.642
K	F_4,40_ = 1.447; *P* = 0.289	F_4,40_ = 1.847; *P* = 0.197	F_1,40_ = 0.389; *P* = 0.547	F_13,40_ = 2.445; *P* = 0.081
Mg	F_4,40_ = 0.434; *P* = 0.781	F_4,40_ = 3.640; *P* = **0.044**	F_1,40_ = 0.283; *P* = 0.606	F_13,40_ = 2.002; *P* = 0.138
Mn	F_4,40_ = 14.108; *P* < **0.001**	F_4,40_ = 44.806; *P* < **0.001**	F_1,40_ = 0.839; *P* = 0.381	F_13,40_ = 7.583; *P* = **0.002**
Na	F_4,40_ = 0.818; *P* = 0.542	F_4,40_ = 1.172; *P* = 0.379	F_1,40_ = 0.043; *P* = 0.840	F_13,40_ = 1.769; *P* = 0.185
Ni	F_4,40_ = 4.382; *P* = **0.026**	F_4,40_ = 0.831; *P* = 0.535	F_1,40_ = 0.063; *P* = 0.807	F_13,40_ = 3.228; *P* = **0.035**
P	F_4,40_ = 0.108; *P* = 0.978	F_4,40_ = 3.321; *P* = 0.056	F_1,40_ = 0.284; *P* = 0.606	F_13,40_ = 1.181; *P* = 0.403
Pb	F_4,40_ = 0.336; *P* = 0.848	F_4,40_ = 0.785; *P* = 0.560	F_1,40_ = 0.526; *P* = 0.485	F_13,40_ = 0.533; *P* = 0.857
Sr	F_4,40_ = 1.317; *P* = 0.378	F_4,40_ = 4.379; *P* = 0.068	F_1,40_ = 0.099; *P* = 0.766	F_13,40_ = 2.850; *P* = 0.127
Ti	F_4,40_ = 4.428; *P* = **0.026**	F_4,40_ = 4.871; *P* = **0.019**	F_1,40_ = 0.025; *P* = 0.878	F_13,40_ = 1.760; *P* = 0.187
Zn	F_4,40_ = 4.291; *P* = **0.028**	F_4,40_ = 24.196; *P* < **0.001**	F_1,40_ = 20.176; *P* = **0.001**	F_13,40_ = 1.488; *P* = 0.268

Statistically significant effects are reported in **bold**

While no significant effects of sampling location, time, depth, and their two-way interactions were found for Cd, Cu, Hg, Pb, and Sr, significant differences were noted for the other TEs (Table 2).

In detail, Mn levels significantly differed among sampling locations, independently of sampling time and depth. In detail, Mn levels measured in S1 were significantly lower than those observed in mussels from S4 (*P* = 0.005) and S5 (*P* = 0.002), while S2 levels were significantly lower than those measured in mussels from S3 (*P* = 0.041), S4 (*P* = 0.003), and S5 (*P* = 0.001). No differences in Mn levels occurred among mussels transplanted in S3, S4, and S5. Interestingly, an overall significant effect of the time of sampling was noted, with a significant increase in Mn levels measured in mussels at *t* = 30 days and *t* = 135 days compared to *t* = 0 days (*P* < 0.001 in both the cases, independently of sampling location and depth). However, a significant decrease in Mn levels was measured at *t* = 166 days and *t* = 196 days compared to *t* = 135 days (*P* < 0.002 in both the cases). In addition, Mn levels measured in mussels transplanted in S3, S4, and S5 at *t* = 135 days were significantly higher than those measured in the same locations at previous time points (*P* < 0.01 in all the cases).

Similar results were obtained for Fe. Levels measured in mussels transplanted in S2 significantly differed from those of all the other sampling locations (*P* < 0.027 in all the cases), independently of sampling time and depth. Overall, Fe levels measured in mussels at *t* = 135 days and 196 days significantly differed when compared to those measured at *t* = 0 day (*P* < 0.030 in both the cases) and *t* = 30 days (*P* < 0.002 in both the cases). Despite statistical analyses returning a significant effect for Location × Time interaction, no clear trend was noted.

Zinc levels measured in mussels transplanted in S5 were significantly higher than those measured in S2 (*P* = 0.027), but they did not differ with respect to other sampling locations. Moreover, Zn levels measured at *t* = 0 days significantly differed from those recorded in the further time points (*P* < 0.004 in all the cases), independently of sampling location and depth. Interestingly, the levels of Zn measured in mussels transplanted at 15-m depth were higher than those measured in conspecifics transplanted at 5-m depth (*P* = 0.001), independently of sampling location and time.

Levels of Ti measured in mussels transplanted in S2 were significantly lower than those measured in S4 (*P* = 0.023) and S5 (*P* = 0.049), but they did not differ from those observed in the other sampling locations. Titanium levels measured at *t* = 135 days were significantly higher than those recorded at *t* = 30 days (*P* = 0.010), independently of sampling location and depth.

Levels of Ni measured in mussels transplanted in S2 were significantly lower from those recorded in S4 and S5 (*P* < 0.05 in both the cases), independently of sampling time and depth. Despite the significant effect of Location × Time interaction, no clear trend was noted.

Lastly, a significant effect of sampling time on Mg, Cr, Al, and As was noted, independently of sampling location and depth. In detail, Mg levels measured in transplanted mussels at *t* = 0 days were significantly lower than those at *t* = 30 days (*P* = 0.043), but no other differences in pairwise comparisons occurred. Levels of Al measured in transplanted mussels at *t* = 166 days were marginally, significantly higher than those measured at *t* = 30 days (*P* = 0.05), independently of sampling location and depth. A similar result was obtained also for As (*P* = 0.009), while As levels measured at *t* = 0 were significantly different compared to those at *t* = 30 (*P* = 0.006) and *t* = 166 days (*P* = 0.014). Levels of Cr measured in transplanted mussels at *t* = 135 days were significantly higher compared to those measured at *t* = 0 (*P* = 0.003) and *t* = 30 days (*P* = 0.001), but were significantly lower than those measured at *t* = 166 (*P* = 0.005) and *t* = 196 days (*P* = 0.037).

## Discussion

The present study shows that active biomonitoring using caged blue mussels represents a suitable approach to monitor levels and trends of different TEs in order to check for the efficacy, safety, and potential drawbacks of ecosystem restoration activities in the Flekkefjord fjord.

### Temporal and spatial variability of TE levels

**Fig. 2:**
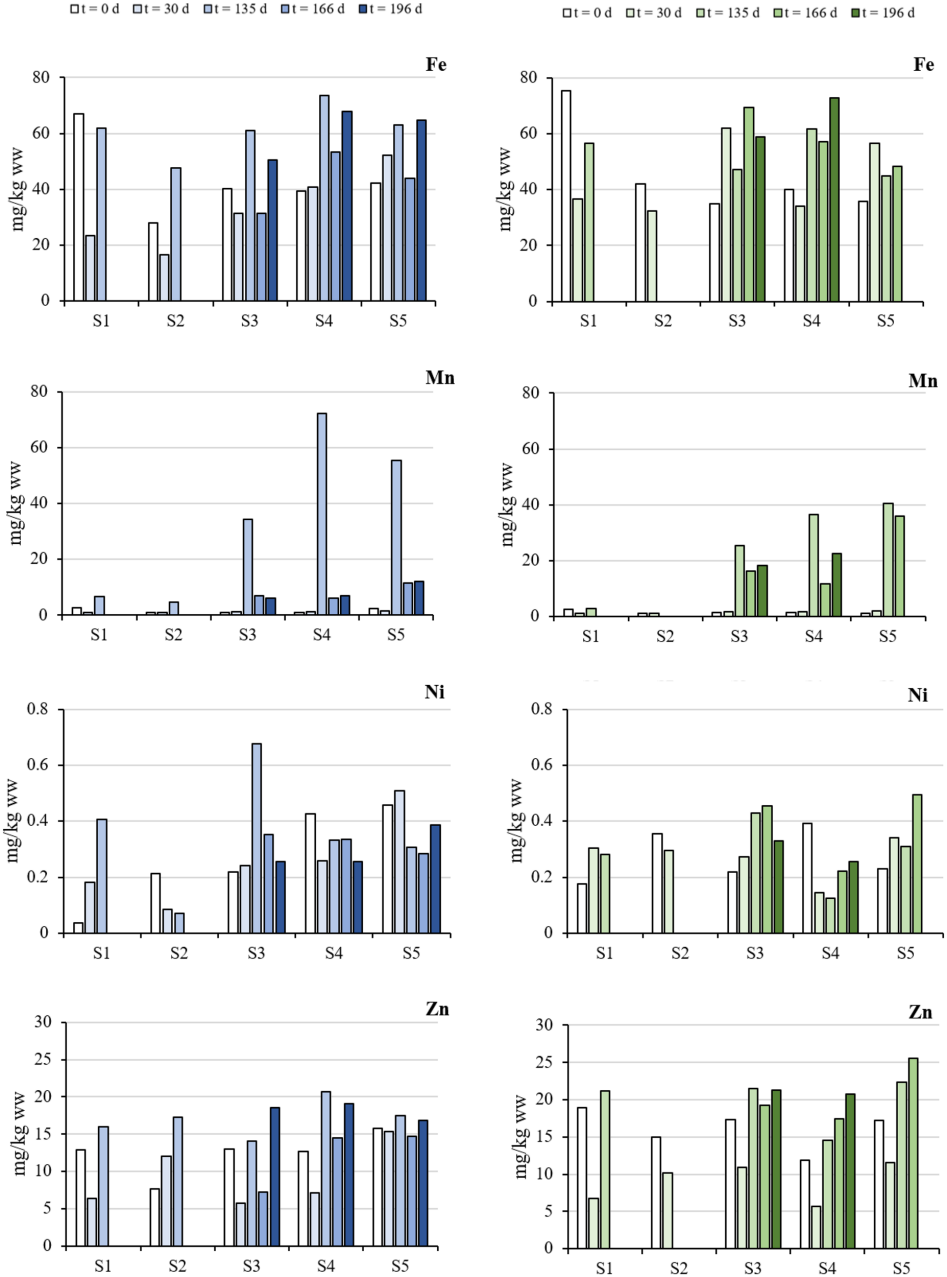
Levels and trends of Fe, Mn, Ni, and Zn measured in blue mussels transplanted in five locations within the Flekkefjord fjord at 5-m (left panels in blue gradient) and 15-m (right panels in green gradient) depth over 6-months biomonitoring activities

Levels of TEs accumulated in tissues of transplanted mussels in the Flekkefjord fjord were generally low before the beginning of the restoration activities. The levels and the fingerprint of contamination were similar to those observed in native and transplanted blue mussels from the city harbour of Kristiansand (Southern Norway), a moderately to severely polluted area by anthropogenic contaminants (Schøyen et al., 2017), as well as to those from native Mediterranean mussels (*Mytilus galloprovincialis*) collected in different areas in the Adriatic Sea (e.g. Esposito et al., 2021; Perošević et al., 2018). However, the implementation of restoration activities caused a time- and location-dependent increase of the concentrations of some specific TEs in mussels. An overall, significant increase of Al, As, Cr, Fe, Mg, Mn, Ti, and Zn concentrations was noted over the whole duration of the biomonitoring, independently of the sampling location. In contrast, the levels of toxic TEs, such as As, Cd, Hg, and Pb, did not follow neither spatial (i.e. location and depth) nor temporal trends, suggesting that they did not increase their bioavailability as a consequence of restoration activities. Previous studies demonstrated that the bioaccumulation and the achievement of a putative steady state was relatively fast for TEs in mussels caged in metal polluted sites, whereby 2-weeks to 1-month deployment seemed to be long enough for stable concentration to be established (Regoli & Orlando, 1994; Schøyen et al., 2017). Overall, the highest levels of all the TEs measured in mussels transplanted to Flekkefjord fjord were found in the winter season, after more than 3 months from the beginning of the monitoring. On one hand, this trend could be partially explained by seasonal variations in metal burdens of mussels due to fluctuations in soft tissue weight related to the reproductive cycle (Farrington et al., 1983; Devier et al., 2005; Perošević et al., 2018). On the other hand, the main contributors to the increase of TE levels in transplanted mussels were accountable to their increased bioavailability due to new inputs from local sources and/or the release from bottom sediments during restoration activities. This hypothesis was supported by the evidence that the levels of Fe, Mn, Ni, and Zn (as well as of Ti, but with unclear trends) differed among sampling locations (Fig. 2). Overall, the concentrations of these TEs were higher in soft tissues from mussels caged at the locations from the inner part of the fjord (S3–S5) compared to those located in its outer part (S1 and S2).

These results were in accordance with previous monitoring studies of sediment contamination performed in this area, where high levels of TEs, mainly heavy metals, were measured in the close proximity of S3, S4, and S5 because of the presence of a naval industry, a landfill and a dismissed tannery, respectively (Haker, 2011; Misund, 2012). Thus, TEs trapped in the sediments from these locations might return bioavailable for mussels as a consequence of sediment resuspension caused by restoration activities. Interestingly, such increase was extremely marked for Fe and Mn in S3, S4, and S5, whose levels showed a significant increased after *t* = 135 days compared to previous time points. During restoration activities, iron-silicomanganese slag was used to build a reservoir of contaminated sediments removed from the bottom of the fjord in close proximity of the S5 location. The increase of both Fe and Mn observed in S5 at *t* = 135 days in mussels transplanted at both 5- and 15-m depth might be due to a release of these TEs from the slag. This hypothesis was supported by a previous analysis that showed the release of Mn from the slag used during restoration activities (COWI, 2018). Furthermore, the massive accumulation of such material on tenuous fjord sediments caused an undersea landslide in the proximity of S5 a week before sampling at *t* = 135 days. Such landslide caused a huge resuspension of sediments and an intense water mixing in the inner part of the fjord, explaining the increase of Fe and Mn, but also of Zn, in S3 and S4. The trend of Fe and Mn levels was similar between mussels transplanted at 5- and 15-m depth. In fact, despite a notable increase of Mn concentrations recorded at *t* = 135 days, with levels up to 73- (at 5-m depth) and 38-fold (at 15-m depth) higher compared to the beginning of the biomonitoring, a further decrease was noted, suggesting a sedimentation of re-suspended sediments and/or particulate matter. A similar trend was already noted for two classes of organic chemicals, namely PCBs and PAHs, in a previous companion study performed in the same area (Parolini et al., 2020a), supporting the hypothesis that the undersea landslide enhanced the bioavailability of organic and inorganic contaminants. Unfortunately, this trend could not be confirmed in S5 because all the mussels caged at 15-m depth died, likely because of the combined effects of contaminant accumulation, mechanical abrasion of gills, reduction in feeding rates, and increased susceptibility to diseases (e.g. Cheung & Shin, 2005; Leverone, 1995).

Although the levels of TEs considered toxic for living organisms (e.g. Pb, Hg, Cr) were low or negligible, the excessive accumulation of Fe and Mn might represent a serious threat for the health status of mussels and, in general, of marine organisms. Fe and Mn are two essential elements playing diverse essential roles in the development and diverse body functions in living organisms, but their excess can result in toxic consequences (Crossgrove & Zheng, 2004; Kádár et al., 2010; Kaur et al., 2017). For instance, the excess of Fe can dysregulate its cellular uptake and transport, while storage proteins are saturated and the intracellular labile Fe pool of weakly bound iron increases (Kádár et al., 2010). When this labile iron reaches critical levels and exceeds the cell antioxidant capacity, it causes the overproduction of reactive oxygen species (ROS), which can induce oxidative stress towards cellular macromolecules such as lipids, proteins, and nucleic acids (Nghia et al., 2002). In addition, abnormal Fe accumulation can cause cytotoxic carboxyl radical synthesis through the Fenton reaction (Valko et al., 2005). Thus, the increased accumulation of Fe in mussels caged at S3–S5 locations might cause the onset of an oxidative stress condition and consequent impairment of organism functions. For instance, a previous laboratory study showed that the exposure to Fe induced an impairment of lysosomal stability in circulating blood cells and lipid peroxidation in the gills of blue mussels (Kádár et al., 2010). Also an excessive exposure to Mn was demonstrated to be detrimental for organisms, arresting cell growth and division (Kaur et al., 2014; Waters et al., 2011), reducing iron transport, and causing neurodegenerative disorders in humans (Kaur et al., 2017). A recent experimental study showed that chronic exposures to low, environmentally relevant concentrations of Mn can disrupt the serotonin system in the blue mussel by reducing the expression of the serotonin transporter (SERT) in the mantle, limiting serotonin cellular transport (Fraser et al., 2018). The expression of SERT is regulated by intracellular concentrations of calcium (Ca^2+^; Seimandi et al., 2013), which can be perturbed by the interaction with Mn and other TEs (Tchounwou et al., 2012). In addition, TEs can interact with diverse functional groups of proteins (e.g. sulfhydryl and amine groups) that could modulate SERT function and limit the cellular transport of serotonin (Disbudak et al., 2002). As serotonin plays a key role in a number of biological functions in mussels, including regulation of metabolism, beating of gill cilia, siphon movement during filtration, relaxation of adductor muscle fibres (Almeida et al., 2003; Carroll & Catapane, 2007; Ram et al., 1999), sexual differentiation, gamete production, and spawning (Gibbons & Castagna, 1984; Ram et al., 1999), Mn exposure might negatively affect mussel health status, fitness, and survival.

### Human health risk assessment

The consumption of local fishery products represents the main pathway of exposure to persistent, bio-accumulative and toxic substances, leading to potential risk for human health (Chiesa et al., 2016, 2018; Panseri et al., 2019). Supposing that native mussels can accumulate a similar amount of contaminants in their soft tissues compared to transplanted conspecifics, the consumption of contaminated mussel could represent a risk for local population. Although all the investigated TEs were accumulated generally in higher concentrations compared to those recorded at the beginning of the biomonitoring, the levels of each single TEs measured in mussels did not represent a hazard for humans. In fact, the concentrations of Cd, Hg, and Pb did not exceed the maximum permissible limits (MPLs) for edibility of bivalve molluscs set by the Commission Regulation No 1259/2011, accounting for 1.0 mg/kg ww for Cd, Hg 0.5 mg/kg ww for Hg, and 1.5 mg/kg ww for Pb, in none of the samples. Similarly, the approach based on THQ suggested a negligible risk for mussel consumption, at least considering singularly every TE. In fact, the calculated THQ was below the unit for all the TEs (Table 3), showing that the level of exposure to individual TEs was lower than the oral reference dose (RfDo) provided by the USEPA (2017). These results suggest that the daily exposure to TEs at concentrations accumulated in the edible part of mussels could not cause negative health effects during a lifetime in human population (Bogdanovic et al., 2014). However, for combined, simultaneous exposure to different TEs, a risk for human consumption can occur also if no single TE exceeds its RfDo (USEPA, 1993). The application of the total HI (Table 3) showed that no additive risk from different TEs could occur in human population of Flekkefjord fjord, as the HI did not exceed the unit in any location and time of sampling.

**Table 3:** Target hazard quotient (THQ) and total hazard index (HI) calculated per sampling location, time, and depth for trace elements whose RfDo was available, namely Al, Fe, Zn, Sr, Mn, Cu, Ni, Pb, Cr, Cd. For Hg the value calculated for methyl-Hg (MeHg) was used

**Location**	**Depth (m)**	**Sampling** **(days)**	**THQ**											**HI**
			**Al**	**Cd**	**Cr**	**Cu**	**Fe**	**Hg**	**Mn**	**Ni**	**Pb**	**Sr**	**Zn**	
	5	*t* = 0	0.0045	0.0075	< 0.0001	0.0001	0.0036	< 0.0001	0.0001	< 0.0001	< 0.0001	0.0005	0.0007	0.0171
	15	*t* = 0	0.0023	0.0067	< 0.0001	0.0002	0.0041	< 0.0001	0.0001	< 0.0001	< 0.0001	0.0008	0.0010	0.0152
	5	*t* = 30	0.0018	0.0070	< 0.0001	0.0001	0.0015	< 0.0001	< 0.0001	< 0.0001	< 0.0001	0.0003	0.0004	0.0112
	15	*t* = 30	0.0032	0.0065	< 0.0001	0.0002	0.0023	< 0.0001	0.0001	< 0.0001	< 0.0001	0.0006	0.0008	0.0137
**S1**	5	*t* = 135	0.0017	0.0069	< 0.0001	0.0001	0.0022	< 0.0001	0.0001	< 0.0001	< 0.0001	0.0003	0.0007	0.0120
	15	*t* = 135	0.0011	0.0066	< 0.0001	0.0003	0.0019	< 0.0001	0.0001	< 0.0001	< 0.0001	< 0.0001	0.0009	0.0110
	5	*t* = 166	0.0036	0.0068	< 0.0001	0.0001	0.0021	< 0.0001	0.0001	< 0.0001	< 0.0001	0.0004	0.0007	0.0138
	15	*t* = 166	0.0052	0.0058	< 0.0001	0.0001	0.0022	< 0.0001	0.0001	< 0.0001	< 0.0001	0.0005	0.0006	0.0146
	5	*t* = 196	0.0026	0.0059	< 0.0001	0.0001	0.0023	< 0.0001	0.0001	< 0.0001	< 0.0001	< 0.0001	0.0008	0.0119
	15	*t* = 196	0.0019	0.0079	< 0.0001	0.0002	0.0019	< 0.0001	0.0001	< 0.0001	< 0.0001	0.0005	0.0009	0.0133
	5	*t* = 0	0.0004	0.0070	< 0.0001	< 0.0001	0.0013	< 0.0001	0.0001	< 0.0001	< 0.0001	0.0006	0.0003	0.0097
	15	*t* = 0	0.0005	0.0115	< 0.0001	0.0001	0.0020	< 0.0001	0.0001	< 0.0001	< 0.0001	0.0002	0.0004	0.0148
	5	*t* = 30	0.0004	0.0054	< 0.0001	0.0003	0.0009	< 0.0001	0.0001	< 0.0001	< 0.0001	< 0.0001	0.0007	0.0078
	15	*t* = 30	0.0008	0.0051	< 0.0001	0.0001	0.0017	< 0.0001	0.0001	< 0.0001	< 0.0001	0.0004	0.0006	0.0089
**S2**	5	*t* = 135	0.0005	0.0063	< 0.0001	< 0.0001	0.0017	< 0.0001	0.0001	< 0.0001	< 0.0001	0.0007	0.0003	0.0096
	15	*t* = 135	0.0007	0.0055	< 0.0001	0.0001	0.0033	< 0.0001	0.0001	< 0.0001	< 0.0001	0.0005	0.0006	0.0108
	5	*t* = 166	0.0006	0.0062	< 0.0001	0.0001	0.0022	< 0.0001	0.0001	< 0.0001	< 0.0001	0.0006	0.0004	0.0102
	15	*t* = 166	0.0004	0.0042	< 0.0001	< 0.0001	0.0018	< 0.0001	0.0001	< 0.0001	< 0.0001	0.0004	0.0003	0.0073
	5	*t* = 196	0.0003	0.0064	< 0.0001	0.0002	0.0028	< 0.0001	0.0001	< 0.0001	< 0.0001	0.0011	0.0008	0.0117
	15	*t* = 196	0.0005	0.0084	< 0.0001	0.0002	0.0030	< 0.0001	0.0001	< 0.0001	< 0.0001	0.0007	0.0006	0.0136
	5	*t* = 0	0.0008	0.0042	< 0.0001	0.0001	0.0033	< 0.0001	0.0004	< 0.0001	< 0.0001	0.0011	0.0009	0.0108
	15	*t* = 0	0.0007	0.0079	< 0.0001	0.0001	0.0031	0.0001	0.0002	< 0.0001	< 0.0001	0.0007	0.0011	0.0140
	5	*t* = 30	0.0006	0.0069	< 0.0001	0.0001	0.0026	< 0.0001	0.0002	< 0.0001	< 0.0001	0.0005	0.0009	0.0118
	15	*t* = 30	n.c	n.c	n.c	n.c	n.c	n.c	n.c	n.c	n.c	n.c	n.c	n.c
**S3**	5	*t* = 135	0.0025	0.0072	< 0.0001	0.0002	0.0033	0.0001	0.0018	< 0.0001	< 0.0001	0.0015	0.0008	0.0174
	15	*t* = 135	0.0013	0.0065	< 0.0001	0.0001	0.0025	< 0.0001	0.0014	< 0.0001	< 0.0001	< 0.0001	0.0012	0.0129
	5	*t* = 166	0.0031	0.0081	< 0.0001	0.0002	0.0040	< 0.0001	0.0039	< 0.0001	< 0.0001	0.0011	0.0011	0.0216
	15	*t* = 166	0.0019	0.0051	< 0.0001	0.0002	0.0033	< 0.0001	0.0020	< 0.0001	< 0.0001	0.0007	0.0008	0.0141
	5	*t* = 196	0.0025	0.0061	< 0.0001	0.0001	0.0034	< 0.0001	0.0030	< 0.0001	< 0.0001	0.0006	0.0009	0.0167
	15	*t* = 196	0.0011	0.0058	< 0.0001	0.0001	0.0024	< 0.0001	0.0022	< 0.0001	< 0.0001	0.0005	0.0012	0.0134
	5	*t* = 0	n.c	n.c	n.c	n.c	n.c	n.c	n.c	n.c	n.c	n.c	n.c	n.c
	15	*t* = 0	n.c	n.c	n.c	n.c	n.c	n.c	n.c	n.c	n.c	n.c	n.c	n.c
	5	*t* = 30	n.c	n.c	n.c	n.c	n.c	n.c	n.c	n.c	n.c	n.c	n.c	n.c
	15	*t* = 30	n.c	n.c	n.c	n.c	n.c	n.c	n.c	n.c	n.c	n.c	n.c	n.c
**S4**	5	*t* = 135	0.0160	0.0057	< 0.0001	0.0001	0.0017	< 0.0001	0.0004	< 0.0001	< 0.0001	0.0005	0.0004	0.0248
	15	*t* = 135	0.0040	0.0046	< 0.0001	0.0002	0.0038	< 0.0001	0.0009	< 0.0001	< 0.0001	< 0.0001	0.0010	0.0145
	5	*t* = 166	0.0019	0.0061	< 0.0001	0.0002	0.0029	< 0.0001	0.0003	< 0.0001	< 0.0001	0.0006	0.0008	0.0128
	15	*t* = 166	0.0029	0.0055	< 0.0001	0.0002	0.0031	0.0001	0.0006	< 0.0001	< 0.0001	0.0013	0.0009	0.0146
	5	*t* = 196	0.0006	0.0107	< 0.0001	0.0001	0.0024	< 0.0001	0.0006	< 0.0001	< 0.0001	0.0006	0.0008	0.0159
	15	*t* = 196	0.0010	0.0070	< 0.0001	0.0001	0.0026	< 0.0001	0.0019	< 0.0001	< 0.0001	0.0005	0.0014	0.0147
	5	*t* = 0	n.c	n.c	n.c	n.c	n.c	n.c	n.c	n.c	n.c	n.c	n.c	n.c
	15	*t* = 0	n.c	n.c	n.c	n.c	n.c	n.c	n.c	n.c	n.c	n.c	n.c	n.c
	5	*t* = 30	0.0006	0.0049	< 0.0001	< 0.0001	0.0025	< 0.0001	0.0001	< 0.0001	< 0.0001	0.0004	0.0008	0.0094
	15	*t* = 30	n.c	n.c	n.c	n.c	n.c	n.c	n.c	n.c	n.c	n.c	n.c	n.c
**S5**	5	*t* = 135	0.0007	0.0039	< 0.0001	0.0001	0.0027	< 0.0001	0.0003	< 0.0001	< 0.0001	0.0005	0.0010	0.0094
	15	*t* = 135	0.0004	0.0051	< 0.0001	0.0002	0.0032	< 0.0001	0.0010	< 0.0001	< 0.0001	< 0.0001	0.0012	0.0110
	5	*t* = 166	0.0008	0.0050	< 0.0001	0.0001	0.0037	< 0.0001	0.0004	< 0.0001	< 0.0001	< 0.0001	0.0010	0.0111
	15	*t* = 166	0.0005	0.0045	< 0.0001	0.0001	0.0039	0.0001	0.0012	< 0.0001	< 0.0001	0.0007	0.0011	0.0122
	5	*t* = 196	0.0007	0.0038	< 0.0001	0.0002	0.0035	< 0.0001	0.0006	< 0.0001	< 0.0001	0.0011	0.0009	0.0110
	15	*t* = 196	n.c	n.c	n.c	n.c	n.c	n.c	n.c	n.c	n.c	n.c	n.c	n.c

*n.c.*, not calculated because concentration data were missing

## Conclusion

The present biomonitoring study demonstrated that the levels of TEs in the Flekkefjord fjord ecosystem were low before the implementation of restoration activities, which directly (e.g. sediment dredging and/or TEs release from slag) or indirectly (i.e. undersea landslide) caused an increase in TE bioavailability and, consequently, their bioaccumulation in mussel soft tissues. In particular, the marked increase in Fe and Mn levels might suggest the release of these TEs from iron-manganese slag used for restoration purposes. However, although this hypothesis was supported by laboratory experiments, it needs to be confirmed by further in-field studies. The levels of TEs, both independently and in mixture, accumulated in mussels did not pose a risk to human health, because they were lower than threshold limits set by the regulations for human consumption. Thus, also supposing that native mussels could accumulate the same amount of TEs than their transplanted conspecifics, a food safety for human consumption of mussels from Flekkefjord fjord can be assumed. Despite these findings, further biomonitoring studies represent a priority in order to check for the progress of restoration activities, paying a special attention to the protection of the ecosystem and human health.

## Data Availability

Data will be made available on reasonable request.
